# STRENGTHENING FAMILY CAREGIVING THROUGH INNOVATIVE TECHNOLOGY SOLUTIONS

**DOI:** 10.1093/geroni/igz038.1648

**Published:** 2019-11-08

**Authors:** David Lindeman, Katherine K Kim

**Affiliations:** 1 University of California Berkeley, Berkeley, California, United States; 2 Betty Irene Moore School Of Nursing and School Of Medicine Public Health Sciences, University Of California Davis, Sacramento, California, United States

## Abstract

Technology has the potential to enhance the repertoire of tools for family caregiving to address the complexities of caring for older adults. There are examples of technology-enabled interventions helping older adults remain independent and safe in their home; easing the financial, physical, and psychological challenges of family caregiving; assisting in the management of chronic illness; improving socialization and support; offering information and resources on a “just in time” basis; and improving the quality of care and quality of life for both older adults and their family caregivers. This session will review eight evidence-based, technology-enabled solutions for family caregivers, including technology solutions that address medication adherence, falls prevention, personal emergency response, remote monitoring, telehealth, dementia tracking, social engagement, and care training. Key drivers for successful application of these interventions (e.g., technology, analytics, user experience design) as well as barriers to scaling (e.g., accessibility, affordability, regulation) will be reviewed.

